# 2-Benzyl-1,3-diphenyl-2,3-dihydro-1*H*-naphtho[1,2-*e*][1,3]oxazine

**DOI:** 10.1107/S1600536809025811

**Published:** 2009-07-08

**Authors:** Yuan Zhang, Yong Hua Li

**Affiliations:** a Ordered Matter Science Research Center, College of Chemistry and Chemical Engineering, Southeast University, Nanjing 211189, People’s Republic of China

## Abstract

In the title compound, C_31_H_25_NO, the oxazine ring adopts a half-chair conformation. The dihedral angles between the phenyl rings and the naphthyl ring system are 70.89 (8), 37.34 (10) and 9.42 (10)°. The crystal structure is stabilized by an aromatic π–π stacking inter­action, with a centroid–centroid distance of 3.879 (3) Å.

## Related literature

For the synthesis and crystal structures of oxazines, see: Alfonsov *et al.* (2007 ▶); Li *et al.* (2008 ▶). For pharmaceutical applications of oxazines, see: Peglion *et al.* (1997 ▶); Xu *et al.* (2004 ▶).
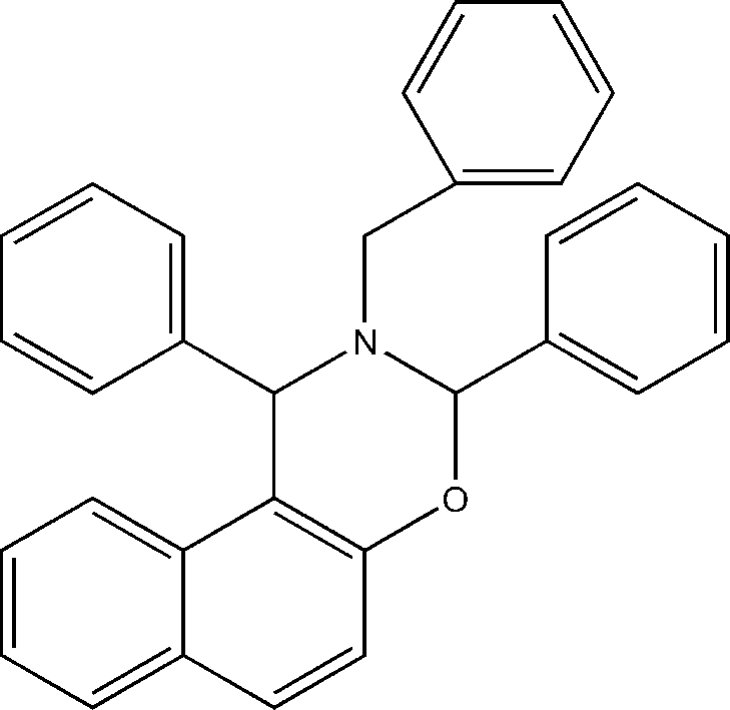


## Experimental

### 

#### Crystal data

C_31_H_25_NO
*M* *_r_* = 427.52
Monoclinic, 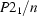

*a* = 9.0605 (18) Å
*b* = 23.475 (5) Å
*c* = 10.634 (2) Å
β = 97.53 (3)°
*V* = 2242.2 (8) Å^3^
*Z* = 4
Mo *K*α radiation
μ = 0.08 mm^−1^
*T* = 293 K
0.20 × 0.20 × 0.20 mm

#### Data collection

Rigaku SCXmini diffractometer
Absorption correction: multi-scan (*SADABS*; Bruker, 2000 ▶) *T*_min_ = 0.984, *T*_max_ = 0.985
18548 measured reflections
4392 independent reflections
2913 reflections with *I* > 2σ(*I*)
*R*_int_ = 0.078

#### Refinement

*R*[*F* ^2^ > 2σ(*F* ^2^)] = 0.058
*wR*(*F* ^2^) = 0.118
*S* = 1.00
4392 reflections
299 parameters
H-atom parameters constrained
Δρ_max_ = 0.16 e Å^−3^
Δρ_min_ = −0.20 e Å^−3^

### 

Data collection: *CrystalClear* (Rigaku, 2005 ▶); cell refinement: *CrystalClear*; data reduction: *CrystalClear*; program(s) used to solve structure: *SHELXS97* (Sheldrick, 2008 ▶); program(s) used to refine structure: *SHELXL97* (Sheldrick, 2008 ▶); molecular graphics: *SHELXTL/PC* (Sheldrick, 2008 ▶); software used to prepare material for publication: *SHELXTL/PC*.

## Supplementary Material

Crystal structure: contains datablocks I, global. DOI: 10.1107/S1600536809025811/rz2345sup1.cif

Structure factors: contains datablocks I. DOI: 10.1107/S1600536809025811/rz2345Isup2.hkl

Additional supplementary materials:  crystallographic information; 3D view; checkCIF report

## Figures and Tables

**Fig. 1.**
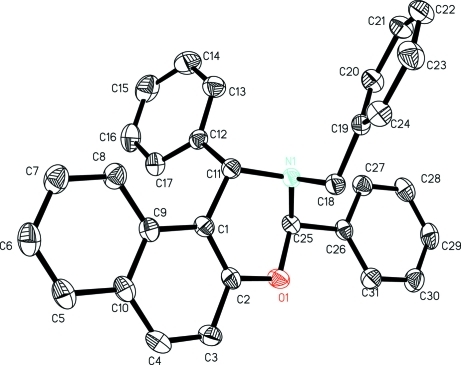
The molecular structure of the title compound, showing the atom-numbering scheme. Displacement ellipsoids are drawn at the 30% probability level. H atoms are omitted for clarity.

## supplementary crystallographic information

### Comment

Continuing efforts have been made to synthesize oxazine compounds (Alfonsov *et al.*, 2007) because they are widely used as antipsychotic agents, antimalarial agents, and serotomin and dopamine receptors agonists (Peglion *et al.*, 1997; Xu *et al.* 2004). We have prepared a novel compound, 2-benzyl-1,3-diphenyl-2,3-dihydro-1*H*-naphtho[1,2-*e*][1,3]oxazine, by the reaction of 2-naphthol, benzaldehyde and phenylmethanamine, and we report in this paper its synthesis and crystal structure. The structures of some closely related compounds have been reported (Alfonsov *et al.*, 2007; Li *et al.*, 2008).

The molecule of the title compound has normal geometric parameters. The oxazine ring adopts a half chair conformation. The dihedral angles formed by the naphthyl ring system with the C12–C17, C19–C24 and C26–C31 phenyl rings are 70.89 (8), 37.34 (10) and 9.42 (10)°, respectively. The crystal structure is stabilized by an aromatic π–π stacking interaction involving the C26—C31 phenyl ring at (x, y, z) and the C5—C10 ring of the naphhtyl ring system at (1+x, y, z), with a centroid to centroid distance of 3.879 (3) Å.

### Experimental

The title compound was one of the products of the reaction between 2-naphthol, phenylmethanamine and an excess amount of benzaldehyde. Benzaldehyde (3.18 g, 0.03 mol) and phenylmethanamine (1.605 g, 0.015 mol) were added to 2-naphthol (2.16 g, 0.015 mol) without solvent under nitrogen atmosphere. The temperature was gradually raised to 120°C in one hour and the mixture was stirred at this temperature for 10 h. The mixture was then treated with ethanol (95%, 20 ml) and cooled to room temperature. The precipitate was filtered and washed with a small amount of ethanol. The title compound was isolated using column chromatography (petroleum ether / ethyl acetate, 2:1 v/v). Single crystals suitable for X-ray diffraction analysis were obtained by slow evaporation of an ethyl acetate solution.

### Refinement

H atoms were positioned geometrically and refined using a riding model, with C—H = 0.93–0.98 Å and *U*_iso_(H) = 1.2*U*_eq_(C).

### Crystal data

**Table:**

C_31_H_25_NO	*F*(000) = 904
*M**_r_* = 427.52	*D*_x_ = 1.266 Mg m^−^^3^
Monoclinic, *P*2_1_/*n*	Mo *K*α radiation, λ = 0.71073 Å
Hall symbol: -P 2yn	Cell parameters from 4392 reflections
*a* = 9.0605 (18) Å	θ = 2.0–26.0°
*b* = 23.475 (5) Å	µ = 0.08 mm^−^^1^
*c* = 10.634 (2) Å	*T* = 293 K
β = 97.53 (3)°	Block, colourless
*V* = 2242.2 (8) Å^3^	0.20 × 0.20 × 0.20 mm
*Z* = 4	

### Data collection

**Table:**

Rigaku SCXmini diffractometer	4392 independent reflections
Radiation source: fine-focus sealed tube	2913 reflections with *I* > 2σ(*I*)
graphite	*R*_int_ = 0.078
CCD_Profile_fitting scans	θ_max_ = 26.0°, θ_min_ = 3.2°
Absorption correction: multi-scan (*SADABS*; Bruker, 2000)	*h* = −11→11
*T*_min_ = 0.984, *T*_max_ = 0.985	*k* = −28→28
18548 measured reflections	*l* = −13→12

### Refinement

**Table:**

Refinement on *F*^2^	Secondary atom site location: difference Fourier map
Least-squares matrix: full	Hydrogen site location: inferred from neighbouring sites
*R*[*F*^2^ > 2σ(*F*^2^)] = 0.058	H-atom parameters constrained
*wR*(*F*^2^) = 0.118	*w* = 1/[σ^2^(*F*_o_^2^) + (0.0185*P*)^2^ + 1.004*P*] where *P* = (*F*_o_^2^ + 2*F*_c_^2^)/3
*S* = 1.00	(Δ/σ)_max_ < 0.001
4392 reflections	Δρ_max_ = 0.16 e Å^−^^3^
299 parameters	Δρ_min_ = −0.20 e Å^−^^3^
0 restraints	Extinction correction: *SHELXL97* (Sheldrick, 2008), Fc^*^=kFc[1+0.001xFc^2^λ^3^/sin(2θ)]^-1/4^
Primary atom site location: structure-invariant direct methods	Extinction coefficient: 0.0082 (6)

### Special details

**Table:**

Geometry. All e.s.d.'s (except the e.s.d. in the dihedral angle between two l.s. planes) are estimated using the full covariance matrix. The cell e.s.d.'s are taken into account individually in the estimation of e.s.d.'s in distances, angles and torsion angles; correlations between e.s.d.'s in cell parameters are only used when they are defined by crystal symmetry. An approximate (isotropic) treatment of cell e.s.d.'s is used for estimating e.s.d.'s involving l.s. planes.
Refinement. Refinement of *F*^2^ against ALL reflections. The weighted *R*-factor *wR* and goodness of fit *S* are based on *F*^2^, conventional *R*-factors *R* are based on *F*, with *F* set to zero for negative *F*^2^. The threshold expression of *F*^2^ > σ(*F*^2^) is used only for calculating *R*-factors(gt) *etc*. and is not relevant to the choice of reflections for refinement. *R*-factors based on *F*^2^ are statistically about twice as large as those based on *F*, and *R*- factors based on ALL data will be even larger.

### Fractional atomic coordinates and isotropic or equivalent isotropic displacement parameters (Å^2^)

**Table:**

	*x*	*y*	*z*	*U*_iso_*/*U*_eq_	
O1	0.56752 (15)	0.05364 (6)	0.18725 (13)	0.0433 (4)	
C25	0.6744 (2)	0.09254 (9)	0.25499 (19)	0.0357 (5)	
H25A	0.6940	0.1227	0.1957	0.043*	
C26	0.8189 (2)	0.06072 (9)	0.29206 (19)	0.0368 (5)	
N1	0.61811 (18)	0.11912 (7)	0.36044 (15)	0.0339 (4)	
C1	0.3834 (2)	0.12590 (8)	0.21580 (19)	0.0355 (5)	
C9	0.2345 (2)	0.14567 (9)	0.18089 (19)	0.0381 (5)	
C11	0.4930 (2)	0.15723 (8)	0.31138 (19)	0.0354 (5)	
H11A	0.4405	0.1672	0.3832	0.043*	
C2	0.4255 (2)	0.07585 (9)	0.16309 (19)	0.0381 (5)	
C3	0.3247 (2)	0.04280 (10)	0.0810 (2)	0.0446 (6)	
H3A	0.3553	0.0086	0.0489	0.054*	
C12	0.5533 (2)	0.21233 (8)	0.2623 (2)	0.0365 (5)	
C8	0.1826 (2)	0.19750 (9)	0.2272 (2)	0.0479 (6)	
H8A	0.2459	0.2190	0.2846	0.058*	
C10	0.1337 (2)	0.11330 (10)	0.0960 (2)	0.0415 (5)	
C20	0.7137 (2)	0.13377 (9)	0.6347 (2)	0.0444 (6)	
H20A	0.7814	0.1453	0.5811	0.053*	
C19	0.5928 (2)	0.10047 (8)	0.58716 (18)	0.0342 (5)	
C5	−0.0124 (2)	0.13407 (11)	0.0593 (2)	0.0521 (6)	
H5A	−0.0790	0.1129	0.0039	0.062*	
C31	0.8413 (2)	0.00538 (9)	0.2547 (2)	0.0453 (6)	
H31A	0.7636	−0.0147	0.2089	0.054*	
C4	0.1826 (2)	0.06123 (10)	0.0491 (2)	0.0463 (6)	
H4A	0.1163	0.0392	−0.0046	0.056*	
C28	1.0728 (3)	0.06368 (11)	0.3912 (2)	0.0540 (6)	
H28A	1.1506	0.0834	0.4378	0.065*	
C27	0.9361 (2)	0.08953 (10)	0.3615 (2)	0.0444 (6)	
H27A	0.9223	0.1266	0.3883	0.053*	
C18	0.5762 (2)	0.07786 (8)	0.45383 (19)	0.0383 (5)	
H18A	0.6375	0.0441	0.4518	0.046*	
H18B	0.4734	0.0666	0.4294	0.046*	
C22	0.6355 (3)	0.13384 (11)	0.8404 (2)	0.0562 (7)	
H22A	0.6495	0.1452	0.9249	0.067*	
C17	0.5268 (2)	0.22873 (10)	0.1367 (2)	0.0468 (6)	
H17A	0.4695	0.2055	0.0785	0.056*	
C7	0.0414 (3)	0.21641 (10)	0.1892 (2)	0.0556 (7)	
H7A	0.0099	0.2508	0.2202	0.067*	
C29	1.0949 (3)	0.00892 (11)	0.3522 (2)	0.0567 (7)	
H29A	1.1876	−0.0083	0.3712	0.068*	
C24	0.4939 (2)	0.08446 (9)	0.6690 (2)	0.0451 (6)	
H24A	0.4115	0.0624	0.6390	0.054*	
C23	0.5154 (3)	0.10079 (11)	0.7951 (2)	0.0559 (7)	
H23A	0.4483	0.0893	0.8493	0.067*	
C30	0.9789 (3)	−0.02034 (11)	0.2850 (2)	0.0554 (6)	
H30A	0.9930	−0.0577	0.2598	0.067*	
C6	−0.0562 (3)	0.18458 (11)	0.1040 (2)	0.0579 (7)	
H6A	−0.1517	0.1981	0.0778	0.069*	
C13	0.6384 (3)	0.24804 (10)	0.3467 (2)	0.0526 (6)	
H13A	0.6572	0.2379	0.4319	0.063*	
C21	0.7349 (3)	0.15011 (10)	0.7604 (2)	0.0516 (6)	
H21A	0.8169	0.1722	0.7910	0.062*	
C16	0.5843 (3)	0.27926 (11)	0.0957 (3)	0.0605 (7)	
H16A	0.5658	0.2897	0.0108	0.073*	
C14	0.6956 (3)	0.29827 (11)	0.3065 (3)	0.0687 (8)	
H14A	0.7526	0.3217	0.3644	0.082*	
C15	0.6686 (3)	0.31363 (11)	0.1813 (3)	0.0690 (8)	
H15A	0.7075	0.3475	0.1542	0.083*	

### Atomic displacement parameters (Å^2^)

**Table:**

	*U*^11^	*U*^22^	*U*^33^	*U*^12^	*U*^13^	*U*^23^
O1	0.0346 (8)	0.0491 (9)	0.0444 (9)	0.0029 (7)	−0.0015 (7)	−0.0122 (7)
C25	0.0343 (12)	0.0402 (12)	0.0318 (12)	−0.0006 (9)	0.0013 (10)	0.0008 (9)
C26	0.0339 (12)	0.0455 (13)	0.0312 (12)	0.0038 (9)	0.0049 (9)	0.0031 (10)
N1	0.0352 (10)	0.0362 (10)	0.0301 (10)	0.0026 (7)	0.0036 (8)	0.0017 (7)
C1	0.0319 (12)	0.0409 (12)	0.0338 (12)	−0.0016 (9)	0.0043 (9)	−0.0002 (9)
C9	0.0306 (12)	0.0461 (13)	0.0376 (12)	−0.0002 (9)	0.0049 (10)	0.0052 (10)
C11	0.0329 (12)	0.0384 (12)	0.0349 (12)	0.0037 (9)	0.0042 (10)	−0.0016 (9)
C2	0.0316 (12)	0.0482 (13)	0.0336 (12)	0.0016 (10)	0.0010 (10)	−0.0002 (10)
C3	0.0443 (14)	0.0490 (14)	0.0397 (13)	−0.0019 (10)	0.0024 (11)	−0.0081 (10)
C12	0.0321 (12)	0.0360 (11)	0.0416 (13)	0.0047 (9)	0.0055 (10)	−0.0007 (10)
C8	0.0348 (13)	0.0492 (14)	0.0592 (16)	0.0011 (10)	0.0040 (11)	0.0008 (12)
C10	0.0321 (12)	0.0559 (14)	0.0362 (13)	−0.0023 (10)	0.0033 (10)	0.0064 (10)
C20	0.0430 (14)	0.0494 (14)	0.0402 (13)	−0.0030 (10)	0.0026 (11)	0.0015 (11)
C19	0.0358 (12)	0.0346 (11)	0.0318 (12)	0.0047 (9)	0.0028 (10)	0.0020 (9)
C5	0.0346 (13)	0.0691 (17)	0.0504 (15)	−0.0039 (12)	−0.0023 (11)	0.0095 (13)
C31	0.0417 (13)	0.0490 (14)	0.0453 (14)	0.0050 (10)	0.0063 (11)	−0.0025 (11)
C4	0.0402 (14)	0.0597 (15)	0.0374 (13)	−0.0096 (11)	−0.0015 (11)	−0.0062 (11)
C28	0.0372 (14)	0.0750 (18)	0.0477 (15)	0.0006 (12)	−0.0022 (11)	0.0039 (13)
C27	0.0371 (13)	0.0496 (14)	0.0456 (14)	0.0006 (10)	0.0016 (11)	−0.0013 (11)
C18	0.0405 (12)	0.0369 (12)	0.0365 (12)	−0.0019 (9)	0.0015 (10)	0.0018 (9)
C22	0.0662 (18)	0.0647 (17)	0.0356 (14)	0.0103 (13)	−0.0016 (13)	−0.0081 (12)
C17	0.0411 (14)	0.0502 (14)	0.0482 (15)	0.0033 (11)	0.0026 (11)	0.0053 (11)
C7	0.0411 (14)	0.0532 (15)	0.0726 (18)	0.0067 (11)	0.0084 (13)	0.0016 (13)
C29	0.0434 (15)	0.0744 (18)	0.0518 (16)	0.0189 (13)	0.0039 (13)	0.0139 (14)
C24	0.0407 (13)	0.0502 (14)	0.0449 (14)	−0.0008 (10)	0.0073 (11)	0.0007 (11)
C23	0.0603 (17)	0.0700 (17)	0.0393 (15)	0.0053 (13)	0.0133 (13)	0.0032 (12)
C30	0.0577 (16)	0.0529 (15)	0.0556 (16)	0.0176 (12)	0.0071 (13)	0.0022 (12)
C6	0.0342 (13)	0.0682 (17)	0.0700 (18)	0.0065 (12)	0.0023 (13)	0.0140 (14)
C13	0.0593 (16)	0.0478 (14)	0.0493 (15)	−0.0060 (12)	0.0017 (12)	−0.0028 (11)
C21	0.0534 (15)	0.0517 (15)	0.0460 (15)	−0.0009 (11)	−0.0074 (12)	−0.0062 (12)
C16	0.0573 (17)	0.0621 (17)	0.0625 (18)	0.0062 (13)	0.0096 (14)	0.0234 (14)
C14	0.074 (2)	0.0496 (16)	0.081 (2)	−0.0176 (14)	0.0049 (16)	−0.0066 (15)
C15	0.0667 (19)	0.0464 (16)	0.095 (2)	−0.0081 (13)	0.0134 (17)	0.0165 (15)

### Geometric parameters (Å, °)

**Table:**

O1—C2	1.381 (2)	C31—C30	1.385 (3)
O1—C25	1.452 (2)	C31—H31A	0.9300
C25—N1	1.434 (2)	C4—H4A	0.9300
C25—C26	1.514 (3)	C28—C29	1.373 (3)
C25—H25A	0.9800	C28—C27	1.379 (3)
C26—C31	1.381 (3)	C28—H28A	0.9300
C26—C27	1.387 (3)	C27—H27A	0.9300
N1—C18	1.472 (2)	C18—H18A	0.9700
N1—C11	1.484 (2)	C18—H18B	0.9700
C1—C2	1.377 (3)	C22—C23	1.371 (3)
C1—C9	1.429 (3)	C22—C21	1.372 (3)
C1—C11	1.515 (3)	C22—H22A	0.9300
C9—C8	1.416 (3)	C17—C16	1.388 (3)
C9—C10	1.418 (3)	C17—H17A	0.9300
C11—C12	1.523 (3)	C7—C6	1.396 (3)
C11—H11A	0.9800	C7—H7A	0.9300
C2—C3	1.411 (3)	C29—C30	1.375 (3)
C3—C4	1.358 (3)	C29—H29A	0.9300
C3—H3A	0.9300	C24—C23	1.383 (3)
C12—C17	1.381 (3)	C24—H24A	0.9300
C12—C13	1.386 (3)	C23—H23A	0.9300
C8—C7	1.364 (3)	C30—H30A	0.9300
C8—H8A	0.9300	C6—H6A	0.9300
C10—C4	1.413 (3)	C13—C14	1.378 (3)
C10—C5	1.417 (3)	C13—H13A	0.9300
C20—C21	1.379 (3)	C21—H21A	0.9300
C20—C19	1.386 (3)	C16—C15	1.371 (4)
C20—H20A	0.9300	C16—H16A	0.9300
C19—C24	1.381 (3)	C14—C15	1.370 (4)
C19—C18	1.503 (3)	C14—H14A	0.9300
C5—C6	1.356 (3)	C15—H15A	0.9300
C5—H5A	0.9300		
			
C2—O1—C25	113.55 (15)	C3—C4—H4A	119.4
N1—C25—O1	112.28 (16)	C10—C4—H4A	119.4
N1—C25—C26	113.27 (16)	C29—C28—C27	120.3 (2)
O1—C25—C26	108.46 (16)	C29—C28—H28A	119.8
N1—C25—H25A	107.5	C27—C28—H28A	119.8
O1—C25—H25A	107.5	C28—C27—C26	120.6 (2)
C26—C25—H25A	107.5	C28—C27—H27A	119.7
C31—C26—C27	118.7 (2)	C26—C27—H27A	119.7
C31—C26—C25	122.93 (19)	N1—C18—C19	113.65 (16)
C27—C26—C25	118.26 (19)	N1—C18—H18A	108.8
C25—N1—C18	113.00 (16)	C19—C18—H18A	108.8
C25—N1—C11	108.72 (15)	N1—C18—H18B	108.8
C18—N1—C11	112.38 (15)	C19—C18—H18B	108.8
C2—C1—C9	118.28 (19)	H18A—C18—H18B	107.7
C2—C1—C11	119.68 (18)	C23—C22—C21	119.7 (2)
C9—C1—C11	122.01 (18)	C23—C22—H22A	120.2
C8—C9—C10	117.80 (19)	C21—C22—H22A	120.2
C8—C9—C1	122.3 (2)	C12—C17—C16	121.1 (2)
C10—C9—C1	119.92 (19)	C12—C17—H17A	119.5
N1—C11—C1	110.28 (16)	C16—C17—H17A	119.5
N1—C11—C12	109.90 (16)	C8—C7—C6	120.5 (2)
C1—C11—C12	114.56 (17)	C8—C7—H7A	119.7
N1—C11—H11A	107.3	C6—C7—H7A	119.7
C1—C11—H11A	107.3	C28—C29—C30	119.5 (2)
C12—C11—H11A	107.3	C28—C29—H29A	120.2
O1—C2—C1	123.19 (18)	C30—C29—H29A	120.2
O1—C2—C3	114.81 (18)	C19—C24—C23	121.0 (2)
C1—C2—C3	122.00 (19)	C19—C24—H24A	119.5
C4—C3—C2	119.7 (2)	C23—C24—H24A	119.5
C4—C3—H3A	120.2	C22—C23—C24	120.1 (2)
C2—C3—H3A	120.2	C22—C23—H23A	120.0
C17—C12—C13	118.0 (2)	C24—C23—H23A	120.0
C17—C12—C11	122.93 (19)	C29—C30—C31	120.4 (2)
C13—C12—C11	119.07 (19)	C29—C30—H30A	119.8
C7—C8—C9	121.2 (2)	C31—C30—H30A	119.8
C7—C8—H8A	119.4	C5—C6—C7	120.4 (2)
C9—C8—H8A	119.4	C5—C6—H6A	119.8
C4—C10—C5	121.7 (2)	C7—C6—H6A	119.8
C4—C10—C9	118.9 (2)	C14—C13—C12	121.1 (2)
C5—C10—C9	119.4 (2)	C14—C13—H13A	119.5
C21—C20—C19	120.9 (2)	C12—C13—H13A	119.5
C21—C20—H20A	119.6	C22—C21—C20	120.3 (2)
C19—C20—H20A	119.6	C22—C21—H21A	119.9
C24—C19—C20	118.1 (2)	C20—C21—H21A	119.9
C24—C19—C18	120.37 (19)	C15—C16—C17	119.6 (2)
C20—C19—C18	121.34 (19)	C15—C16—H16A	120.2
C6—C5—C10	120.7 (2)	C17—C16—H16A	120.2
C6—C5—H5A	119.7	C15—C14—C13	120.0 (3)
C10—C5—H5A	119.7	C15—C14—H14A	120.0
C26—C31—C30	120.3 (2)	C13—C14—H14A	120.0
C26—C31—H31A	119.8	C14—C15—C16	120.2 (2)
C30—C31—H31A	119.8	C14—C15—H15A	119.9
C3—C4—C10	121.2 (2)	C16—C15—H15A	119.9
